# Molecular Characterization and Antibiotic Resistance of Broad‐Spectrum *β*‐Lactamase‐Producing Enterobacterales in Urinary Tract Infections in Libreville, Gabon

**DOI:** 10.1155/ijm/3553027

**Published:** 2026-06-16

**Authors:** Jean Fabrice Yala, Ornella Zong Minko, Rolande Mabika Mabika, Franck Mounioko, Elvire Anita Mbongo-Kama, Annicet Clotaire Dikoumba, Alain Souza

**Affiliations:** ^1^ Medical Analysis Research Unit, Bacteriology Laboratory, Interdisciplinary Center of Medical Research of Franceville (CIRMF), Franceville, Gabon; ^2^ Agrobiology Research Unit, Molecular and Cellular Biology Laboratory, Masuku University of Science and Technology (USTM), Franceville, Gabon; ^3^ Vector Systems Ecology Unit, Interdisciplinary Center of Medical Research of Franceville (CIRMF), Franceville, Gabon; ^4^ Bacteriology Unit, Medical Analysis Laboratory, Army Training Hospital Omar Bongo Ondimba, Libreville, Gabon; ^5^ Agrobiology Research Unit, Laboratory of Electrophysiology and Pharmacology, Masuku University of Science and Technology (USTM), Franceville, Gabon

**Keywords:** *β*-lactamase, Enterobacterales, gabon, multidrug-resistant, urogenital infection

## Abstract

**Background:**

The effectiveness of antibiotic therapy for urogenital tract infections (UTIs) is increasingly compromised by the global dissemination of extended‐spectrum *β*‐lactamase‐producing Enterobacterales (ES*β*L‐En). This study aimed to assess the involvement of broad‐spectrum *β*‐lactamase‐producing Enterobacterales (BS*β*L‐En) in UTIs and to establish their antibiotic susceptibility profile.

**Methods:**

A cross‐sectional study was conducted from October to December 2019 at the Army Training Hospital Omar Bongo Ondimba. All patients suspected of having a UTI were included. BS*β*L‐En isolates were recovered using MacConkey agar supplemented with cefotaxime. Phenotypic screening for *β*‐lactamase (ES*β*L, AmpC, and carbapenemases) using specific phenotypic confirmatory disc diffusion test. Antimicrobial susceptibility was determined using the disk diffusion method and minimum inhibitory concentration (MIC) assays. The presence of resistance genes (*bla*_CTX-M_, *bla*_TEM_, *bla*_CARBA_, *bla*_IMP_, *mcr-1* and *mcr-2*) was investigated by polymerase chain reaction.

**Results:**

A total of 295 patients were enrolled, among whom 18.3% (54/295) had UTIs. Of the isolated uropathogens 74.1% were BS*β*L‐En. The predominant species were *Enterobacter cloacae* (8/54, 14.8%), *Escherichia coli* (6/54, 11.1%) and *Kluyvera* spp (5/54, 9.3%). Among 40 BS*β*L‐En isolates, 87.5% (*n* = 35/40) were confirmed by molecular analysis. Notably 48.5% (17/35) harbored at least two *bla* genes, simultaneously. Fourteen isolates carried the *bla*_CARBA_ and *bla*_IMP_ genes, which in combination with the *bla*_CTX-M_ and *bla*_TEM_. About 40.0% of the isolates (14/35) carried the *bla*_CTX-M_, 8.6% had the *bla*_TEM_ (3/35), and 2.9% had the *bla*_IMP_ (*n* = 1/35). All urinary isolates were multidrug‐resistant (MDR), exhibiting high levels of *β*‐lactamase production, with resistance rates of 81.6*%* ± 0.16*%* for first‐line antibiotics (e.g., *β*‐lactams, fluoroquinolones) and 61.7*%* ± 0.04*%* for last‐resort antibiotics (e.g., imipenen, ertapenem, and colistin). The prevalence of carbapenem resistance genes was 44.1%, whereas no colistin resistance genes (*mcr*‐1 and *mcr*‐2) were detected.

**Conclusion:**

This study highlights a high prevalence of multidrug‐resistant uropathogens producing BS*β*L, with significant resistance to both first‐line and last‐resort antibiotics.

## 1. Background

Urinary tract infections (UTIs) comprise a heterogeneous group of infections affecting the urinary tract and its associated structures [1]. They are among the most common infectious diseases in both hospital and community settings [2]. In 70% to 95% of cases, the main uropathogens are *Escherichia coli* (*E. coli*) followed by *Klebsiella pneumoniae* and *Proteus mirabilis*. These pathogens are generally derived from the intestinal microbiota [3].

Several community‐based studies have reported that *E. coli* (46.4%–74.2%) followed by *Klebsiella* spp (6.0%–13.45%), *Enterococcus* spp (5.3%–9.54%), and *Proteus* spp (4.7%–11.9%) [4]. The global incidence of UTIs has been estimated at approximately 1.6% [5]. UTIs primarily affect women, with a recurrence risk of 25%–35% per year, whereas recurrence in men is considerably lower [2]. However, in children aged 2 to 24 months, this tendency seems to be reversed in both sexes [6]. Depending on the person′s age, the frequency and recurrence of UTIs can be taken as signs of health complications or/and more severe underlying pathologies. UTIs constitute significant public health problems as they can lead to permanent kidney damage and diabetes [7]. In women, recurrent infections may also affect the reproductive system and contribute to secondary infertility [8].

The management of UTIs is based on CBEU, often following empirical antibiotic therapy or inappropriate self‐medication practices [9]. The increasing prevalence of ES*β*L‐En has become a major concern, as these organisms frequently harbor multiple resistance determinants, thereby limiting therapeutic options [10]. Treatment options for UTIs cause to ES*β*Ls‐producing Enterobacterales include nitrofurantoin, fosfomycin, fluoroquinolones, cefoxitin (FOX), piperacillin‐tazobactam (TZP), carbapenems, ceftazidime‐avibactam, ceftolozane‐tazobactam, and aminoglycosides. Depending on susceptibility results, carbapenem‐sparing regimens may be considered for mild to moderate UTIs caused by ES*β*L‐En [10]. For carbapenem‐resistant Enterobacterales (CRE), therapeutic options include ceftazidime‐avibactam, colistin, polymixin B, fosfomycin, aztreonam, aminoglycosides, and tigecycline [10].

Multidrug‐resistant (MDR) bacteria increasingly threaten the efficacy of last‐resort antibiotics, including carbapenems and colistin [11]. Several studies have reported alarming resistance rates of up to 60% to carbapenems and 40% for colistin among *E. coli* strains [12, 13]. Given their high frequency and clinical impact of these infections, continuous monitoring of antibiotic resistance profile among BS*β*L‐En is essential.

In Gabon, a Central African country, previous studies have described epidemiology of uropathogens in Libreville and Franceville, as well as susceptibility pattems [14, 15]. These studies showed that *E. coli* isolate were the predominant among Enterobacterales species responsible for UTIs, with resistance rate of approximately 65% to penicillin and 45% to aminopenicillin [14, 15]. However, data specifically focusing on BSBL‐En‐associated UTIs remain limited.

Therefore the aim of this study was to investigate and characterize BS*β*L‐En involved in UTIs among inpatients and outpatients at the Army Training Hospital Omar Bongo Ondimba (HIAOBO) in Libreville, Gabon, to determine their susceptibility profiles to first‐line antibiotics, and to perform the phenotypic and genotypic characterization of resistance to last‐line antibiotics.

## 2. Methods

### 2.1. Study Design

This cross‐sectional study was conducted at the HIAOBO in Libreville, Gabon. It is a 3rd generation university hospital, with several departments (intensive care, surgery, medicine, pediatrics, emergency, gynecology, etc.) and a medical analysis laboratory. The laboratory offers a range of services for outpatients and inpatients. Urine samples were collected from patients with a suspected UTI and admitted to the various departments of the hospital, as well as outpatients who came to perform a cytobacteriological examination of their urine. The study was conducted from October 28 to December 28, 2019.

Patients, of all genders and ages, suspected of UTIs, were randomly recruited. The main inclusion criterion was that patients agreed to participate in the study and those who had a prescription for ordered for urine culture and sensitivity analysis. Patients undergoing other medical examinations were excluded from the study. The demographic and clinical data of the patients were recorded. The processing of the isolates after culture was carried out at the Laboratory of Molecular and Cellular Biology (LABMC), Department of Biology of the Masuku University of Sciences and Technology (USTM).

Written informed consent was obtained from patients, or parents or guardians of participating minors. For each participant, characteristics such as demographic (age, sex), source (outpatient, or inpatient), and services (neonatal, medicine, emergency ambulatory service, pediatrics, intensive care, cardiology) were collected on a standard survey form.

### 2.2. Isolation and Identification of Bacteria

The 1st jets of the various urine samples were collected in sterile jars. Then 10 *μ*L were collected using calibrated loops which were subsequently streaked tight, parallel on the surface of CLED agar and then MacConkey agar supplemented medium with cefotaxime (McC‐CTX) concentrated at 2 *μ*g/mL to detect ES*β*L‐En or cephalosporinase (AmpC) producers (AmpC‐En). After incubation at 37°C for 18–24 h, the various presumptive colonies cefotaxime‐resistant isolates were obtained were obtained on McC‐CTX and further subcultured in soft heart–brain agar (BCC) and before then stored at 4°C for subsequent identification. All colonies obtained were then screened by orientation tests: gram staining, oxidase, and catalase tests. Identification of the bacteria was performed by the Gallery of Analysis of Identification Profiles (API) 20E (Biomerieux, S.A, France).

### 2.3. Phenotypic Detection of the Production of Broad‐Spectrum *β*‐lactamases (BS*β*L): ES*β*L, AmpC and Carbapenemase

Phenotypic detection of BS*β*L production (ES*β*L, AmpC, and carbapenemases) on the various selected bacteria was performed by a standard antibiogram using the disk diffusion method.

### 2.4. Detection of ES*β*L‐Producing Strains

The detection of ES*β*L‐producing strains was carried out using two diffusion methods: the synergy test and antimicrobial susceptibility testing interpretation. The double‐disk synergy test was carried out using third‐generation cephalosporin ceftazidime (CAZ), a fourth‐generation cephalosporin cefepime (FEP), monobactam aztreonam (ATM) and amoxicillin and clavulanic acid (AMC). The appearance of a characteristic “champagne cork” or “keyhole” (synergy) effect between the *β*‐lactam antibiotic disks and the clavulanic acid disk on Mueller–Hinton agar was interpreted as indicative of ES*β*L production [16, 17]. Interpretation of antimicrobial susceptibility testing was performed according to the method described by Robin et al. [17]. The procedure and interpretive criteria of the disk diffusion test were carried out in accordance with the recommendations of the Antibiogram Committee of the French Society of Microbiology (CA‐SFM) 2019 V2.0.

### 2.5. Detection of Strains‐Producing AmpC

Two methods were used to characterize AmpCs *β*‐lactamase production: the disk diffusion method and interpretation of the antimicrobial susceptibility test.

The diffusion‐on‐disk method consisted of measuring inhibition zone diameters around FOX disks (FOX, 30 *μ*g) and FEP disks (FEP, 30 *μ*g), which were used for the screening of AmpC‐producing strains [18, 19]. Isolates exhibiting an inhibition zone diameter ≤ 18 mm for FOX and ≥ 27 mm for FEP, in the absence of a synergy effect, were classified as AmpC *β*‐lactamase producers.

### 2.6. Detection of Strains‐Producing Carbapenemases

Phenotypic detection of carbapenemase production was inferred from the antimicrobial susceptibility testing profile, in accordance with the interpretative approach described by Nordmann et al. [20] (Table 1). This screening strategy is widely used in resource‐limited settings as a first‐line approach for the presumptive detection of carbapenemase‐producing Enterobacterales when confirmatory rapid tests are not available.

**Table 1:** Resistance phenotypes resulting from the expression of the main carbapenemases reported in Enterobacterales without or with extended‐spectrum *β*‐lactamases (ES*β*Ls).

	AMX	AMC	TZP	CTX	CAZ	IMP	ETP	ATM	Reference
KPC	R	S/I	R	R	R	S/I/R	I/R	R	[20]
KPC + ES*β*L	R	I/R	R	R	R	I/R	I/R	R	
IMP/VIM/NDM	R	R	I/R	R	I/R	S/I/R	I/R	S	
IMP/VIM/NDM + ES*β*L	R	R	I/R	R	R	I/R	R	R	
OXA‐48/OXA‐181	R	R	S/I/R	S/I	S	S/I	S/I	S	
OXA‐48/OXA‐181 + ES*β*L	R	R	I/R	R	R	I/R	I/R	R	

Abbreviations: AMC, amoxycillin‐clavulanic acid; AMX, amoxycillin; ATM, aztreonam CAZ, ceftazidime; CTX, cefotaxime; ETP, ertapenem; IMP, imipenem; TZP, piperacillin‐tazobactam;.

Carbapenemase production was suspected when exhibited reduced susceptibility or resistance to key *β*‐lactam antibiotics, including amoxicillin, AMC, TZP, cefotaxime, ceftazidime, imipenem, ertapenem, and aztreonam, based on their inhibition zone diameters.

### 2.7. Susceptibility Profiles of BS*β*L‐En to the Usual and Latest Line Antibiotics

Antimicrobial susceptibility testing was performed using the ATB UR EU (08) gallery (bioMérieux, Marcy l′Étoile, France), according to the manufacturer′s instructions. A total of 21 antibiotics were tested: ampicillin (AMP); amoxicillin + clavulanate, ticarcillin (TIC), piperacillin (PIC), piperacillin + tazobactam, cefolatine, cefixim, cefoxitin, cefotaxime, ceftazidim, FEP, imipenem, gentamicin, tobramycin, amikacin, ofloxacin, ciprofloxacin, levofloxacin, co‐trimoxazole, fosfomycin, and nitrofurantoin. The galleries were inoculated with bacterial suspensions adjusted to 0.5 McFarland and incubated at 37°C for 18–24 h. Results were interpreted by assessing the turbidity of the wells, in accordance with the manufacturer′s guidelines. Isolates were categorized as susceptible, intermediate, or resistant. MDR strains were defined as those exhibiting acquired nonsusceptibility to at least one agent in three or more antimicrobial categories.

In this study, all tested antibiotics were considered first‐line agents, except for imipenem. Imipenem, along with ertapenem and colistin, was classified as a last‐resort antibiotic for the treatment of UTI in this study. Susceptibility was determined by the reference method to ertapenem according to Kirby‐Bauer, the broth microdilution for colistin. All results were interpreted according to the recommendations of the CA‐SFM, 2019, Version 2.0. Each test was performed in quadruplicate (*n* = 4).

### 2.8. Molecular Typing of BS*β*L‐En Genes and Search for Resistance Genes to Colistin and Carbapenems

#### 2.8.1. DNA Extraction

The extraction of bacterial DNA was done by heat treatment. Briefly, colonies were diluted in microtubes containing 200 *μ*L ultrapure water and homogenized water. The tubes were incubated in a dry water bath at 100°C for 10 min. The obtained lysates obtained were centrifuged at 12,000 rpm for 8 min. The resulting supernatant were transferred into new microtubes and served as matrix for DNA.

#### 2.8.2. Characteristics of the Genes and Study Conditions

Molecular typing of ES*β*L (*bla*_CTX-M_ and *bla*_TEM,_) as well as the search for carbapenems (*bla*_CARBA_ and *bla*_IMP_) and colistin (*mcr*1 and *mcr*2) resistance genes were carried out by the polymerase chain reaction (PCR) amplification on all strains. The sequences of the different primers and the sizes of the genes are recorded in Table 2.

**Table 2:** Primers used for PCR amplification of genes from *β*‐lactamases and resistance to carbapenems and colistin.

	Primer	Primers ^′^sequences (5 ^′^3 ^′^)	Size (Kb)	Genes	References
*β*‐lactamases	CTX‐M‐F	ATGGTTAAAAAATCACTGC	864	*Bla*_CTX-M_	[16, 21]
	CTX‐M‐R	TTGGTGACGATTTTAGCCGC			
	TEM‐F	ATTCTTGAAGACGAAAGGGC	1150	*Bla*_TEM_	[16, 21]
	TEM‐R	ACGCTCAGTGGAACGAAAAC			

Carbapenemases	IMP‐F	GAAGGCGTTTATGTTCATAC	587	*Bla*_IMP_	[22]
	IMP‐R	GTACGTTTCAAGAGTGATGC			
	CARBA‐F	AATGGCAATCAGCGCTTC	586	*Bla*_CARBA_	[22]
	CARBA‐R	GGGGCTTGATGCTCACT			

Colistin	Mcr‐1‐F	AGTCCGTTTGTTCTTGTGGC	1497	*Mcr1*	[23]
	Mcr‐1‐R	AGATCCTTGGTCTCGGCTTG			
	Mcr‐2‐F	CAAGTGTGTTGGTCGCAGTT	576	*Mcr2*	[23]
	Mcr‐2‐R	TCTAGCCCGACAAGCATACC			

Abbreviation: CARBA, Carbapenemase; CTX‐M, Cefotaximase from Munich; F, Foward; R, Reverse; Kb, Kilobase; mcr, mobilized colistin resistance; TEM, Temoniera; IMP, Imipenemase metallo‐*β*‐lactamase.

The single or multiplex PCR reactions were carried out in a thermocycler (Bio‐RAD, T100TM, USA) in a final reaction volume of 20 *μ*L containing 2 *μ*L of DNA from the bacterial clones and 18 *μ*L of the PCR mix, composed of master mix 1X (AmpliTaqGoldTM 360, Applied Biosystems Thermo Fisher, Luthhuania) and primers (0.5 *μ*M). The different amplification programs were as follows: an initial denaturation step at 95°C for 15 min followed by a 32‐cycle step consisting of denaturation at 94°C for 1 min, hybridization at 55°C (*bla*_TEM_ and *bla*_CTX-M_) or 58°C (*bla*_IMP_, *bla*_CARBA_, and *mcr-*1/2) for 1 min and elongation for 1 min at 72°C. Final elongation was done at 72°C for 10 min. After amplication PCR‐amplified DNA fragments were separated by electrophoresis on a 1.5% (w/v) agarose gel prepared in 1X Tris‐borate‐EDTA (TBE) solution containing ethidium bromide and visualized under an ultraviolet transilluminator (LABOLAN, ECX‐15 M, Spain).

### 2.9. Ethical Considerations

The study was approved on September 15, 2019 by the ethical committee of the hospital structure in accordance with the recommendations of the decree PROT N° 0020/2015/SG/CNE of the Gabonese National Ethics Committee for Research and the Ministry of Health. The study protocol was approved and authorized by the Management of the HIAOBO and the Head of the Medical Analysis Laboratory, Bacteriology Unit. Urine samples were collected from all types of patients after obtaining their written and informed consent for adults, and from the guardians of minor participants.

### 2.10. Statistical Analysis

All the data collected during this study were entered and managed using Microsoft Excel 2010 (Microsoft Corporation, 2010). Statistical analyses were performed using R software (Version 3.2.2)…

The chi‐squared test allowed us to assess possible significant differences between the various sociodemographic parameters. The kappa coefficient was applied to measure the level of agreement between the presence of different *β*‐lactamase encoding genes and the observed antibiotic resistance phenotypes, with interpretation based on a 95% confidence interval [24]. All these statistical tests were two‐tailed, and a *p* value < 0.05 was considered statistically significant.

## 3. Results and Discussion

### 3.1. Results

#### 3.1.1. Demographics and Prevalence of UTIs

In this study, 295 patients were enrolled. The study population was predominantly composed of outpatients (93.2%), whereas hospitalized patients accounted for 6.8%, of whom 70.0% were admitted to medical department. The sex ratio was 1.23, with a higher proportion of females (55.2%) than males (44.8%) (Table 3). Furthermore, the average age was 35.6 ± 17.4 years old. The most represented age group consisted of patients aged 21 to 30 years (29.2%). The rate of infections caused by BS*β*L‐En was 18.31% (54/295). The demographic characteristics of the study population are summarized in Table 3.

**Table 3:** Sociodemographic characteristics of the study population.

	Hospitalized patients	Outpatients	Total	Hospitalized patients	Outpatients	Total	*p*
				UTI‐presumptive BS*β*L			
	*n* = 20 (6.8)	*n* = 275 (93.2)	*n* = 295 (100)	*n* = 2 (0.7)	*n* = 52 (17.6)	*n* = 54 (18.3)	
	*n* (%)	*n* (%)	*n* (%)	*n* (%)	*n* (%)	*n* (%)	
**Gender**							
Female	10 (50.0)	153 (55.6)	163 (55.2)	1 (50.0)	37 (71.2)	38 (70.4)	0.6245
Male	10 (50.0)	122 (44.4)	132 (44.8)	1 (50.0)	15 (28.8)	16 (29.6)	
**Gender ratio F/M**	1	1.3	1.23	1.0	2.5	2.4	
**Age (years)**							
0–10	3 (15.0)	18 (6.5)	21 (7.1)	—	1 (1.9)	1 (100)	0.0104
11–20	2 (10.0)	33 (12.0)	35 (11.5)	—	7 (13.5)	7 (13.0)	
21–30	3 (15.0)	83 (30.2)	86 (29.2)	1 (50.0)	14 (26.9)	15 (27.8)	
31–40	—	61 (22.2)	61 (20.7)	—	11 (21.1)	11 (20.4)	
41–50	3 (15.0)	32 (11.6)	35 (11.9)	—	7 (13.5)	7 (13.0)	
51–60	3 (15.0)	23 (8.4)	26 (8.8)	1 (50.0)	5 (9.6)	6 (11.1)	
61 or more	6 (30.0)	25 (9.1)	31 (10.5)	—	8 (15.4)	8 (14.8)	
**Hospital unit**							
External	—	275 (100)	275 (93.2)	—	5 (100.0))	52 (96.3)	? 2.2.10^-6^
Neonatal	2 (10.0)	—	2 (0.7)	—	—	—	
Medicine	14 (70.0)	—	14 (4.8)	2 (100.0)	—	2 (3.7)	
EAS	1 (5.0)	—	1 (0.3)	—	—	—	
Pediatrics	1 (5.0)	—	1 (0.3)	—	—	—	
Intensive care	1 (5.0)	—	1 (0.34	—	—	—	
Cardiology	1 (5.0)	—	1 (0.3)	—	—	—	

Abbreviation: BS*β*L, Broad‐Spectrum *β*‐Lactamase; EAS, Emergency Ambulatory Service; UTI, Urinary Tract Infections.

The results in Table 3 highlight that the majority of the population infected by presumptive BS*β*L uropathogens was made up of outpatients (17.6%), and a minority of minority were hospitalized patients (0.7%). The chi‐squared test showed a significant difference regarding the origin of the patients (*p* < 2.2.10^−6^). The gender ratio of the infected population was 2.4 with a higher rate of women (70.4%). The most affected age groups are 21 to 30 years and 31 to 40 years with prevalence rates of 27.8% and 20.4%, respectively. However, the chi‐squared test did not show any significant difference between the two genders (*p* = 0.6245), whereas there was a significant difference between age groups (*p* = 0.0104).

#### 3.1.2. Distribution and Frequency of Presumptive Isolates Containing Broad‐Spectrum *β*‐Lactamases Producing Enterobacterales (BS*β*L‐En)

Presumptive Gram‐negative BS*β*L‐En were isolated from 54 urine specimens. Their distribution and rates are presented in Table 4.

**Table 4:** Etiologies of urinary tract infections (UTIs).

	Number of isolates (N)	Percentage %
Gram‐negative Bacilli	44	81.5
Enterobacteria	40	74.1
*Citrobacter koseri/farmeri*	1	1.9
*Enterobacter cloacae*	8	14.8
*Enterobacter gergovia*	1	1.9
*Enterobacter sakasakii*	2	3.7
*Escherichia coli*	6	11.1
*Klebsiella oxytoca*	1	1.9
*Klebsiella pneumoniae*	4	7.4
*Kluyvera* spp	5	9.3
*Proteus mirabilis*	4	7.4
*Raoultella ornithinolytica*	1	1.9
*Serratia fonticola*	2	3.7
*Serratia marcescens*	1	1.9
*Serratia odorifera 1*	3	5.6
*Yersinia intermedia*	1	1.9
Nonfermentative Gram‐negative	4	7.4
*Pseudomonas* spp	3	5.5
*Acinetobacter baumannii*	1	1.9

The results in Table 4 showed that of the 54 bacterial isolates, Gram‐negative bacilli were responsible for most UTIs (81.5%). Among the Gram‐negative bacilli, Enterobacterales were mainly responsible for UTIs (74.7%) followed by nonfermentative Gram‐negative bacilli (7.4%). Gram‐negative bacteria included 44 bacterial strains; 40 were BS*β*L‐En (9 genera, 14 species), whereas 4 were nonfermentative bacilli isolates (2 genera, 4 species). The three predominant Gram‐negative species were *Enterobacter cloacae* (14.8%), *E. coli* (11.1%), and *Kluyvera* spp (9.3%) (Table 4).

#### 3.1.3. Characterization of BS*β*L‐En and Phenotypic Resistance Profiles

The phenotypic tests for the confirmation of BS*β*L‐En identified 40 (100.0%) producers, of which 4 (10.0%) producers of carbapenemases, 6 (15.0%) ES*β*L‐En, 7 (17.5%) AmpC producers and 23 (57.5%) combined producers of AmpC/ES*β*L as presented in (Table 5). The majority of ES*β*L‐En were isolated from women (69.0%; 20/29) compared to men (31.0%; 9/29). In addition, the dissemination of these BS*β*L‐En mainly came from outpatients 93.1% (27/29).

**Table 5:** Frequency of BS*β*L‐producing Enterobacterales and their antibiotic susceptibility profiles.

	BS*β*L	Usual antibiotics																				2nd line ATB therapy		
	*n* (%)	AMP	AMC	TIC	PIC	TZP	CFT	CFM	CXT	CTX	CAZ	FEP	GEN	TOB	AKN	LVX	OFL	CIP	TSU	FOS	FUR	IMI	ERT	CS
**AmpC**	**7 (17.5)**	**7 (100)**	**7 (100)**	**7 (100)**	**7 (100)**	**4 (57.1)**	**7 (100)**	**7 (100)**	**7 (100)**	**7 (100)**	**6** **(85.7)**	**5 (71.4)**	**6 (85.7)**	**3 (42.9)**	**3 (42.9)**	**3 (42.9)**	**4 (57.1)**	**4 (57.1)**	**4 (57.1)**	**5 (71.4)**	**5 (83.3)**	**5 (83.3)**	**4 (57.1)**	**2 (28.6)**
*E. cloacae*	3 (42.8)	3 (100)	3 (100)	3 (100)	3 (100)	1 (33.3)	3 (100)	3 (100)	3 (100)	3 (100)	2 (66.7)	1 (33.3)	2 (66.7)	0 (0.0)	0 (0.0)	0 (0.0)	1 (33.3)	1 (33.3)	1 (33.3)	2 (66.7)	2 (66.7)	1 (33.3)	1 (33.3)	1 (3.3)
*E. gergoviae*	1 (14.3)	1 (100)	1 (100)	1 (100)	1 (100)	1 (100)	1 (100)	1 (100)	1 (100)	1 (100)	1 (100)	1 (100)	1 (100)	1 (100)	1 (100)	1 (100)	1 (100)	1 (100)	1 (100)	1 (100)	1 (100)	1 (100)	1 (100)	0 (0.0)
*E. coli*	2 (28.6)	2 (100)	2 (100)	2 (100)	2 (100)	1 (50.0)	2 (100)	2 (100)	2 (100)	2 (100)	2 (100)	2 (100)	2 (100)	2 (100)	2 (100)	1 (50.0)	1 (50.0)	1 (50.0)	1 (50.0)	1 (50.0)	1 (50.0)	2 (100)	2 (100)	0 (0.0)
*P. mirabilis*	1 (14.3)	1 (100)	1 (100)	1 (100)	1 (100)	1 (100)	1 (100)	1 (100)	1 (100)	1 (100)	1 (100)	1 (100)	1 (100)	1 (100)	1 (100)	1 (100)	1 (100)	1 (100)	1 (100)	1 (100)	1 (100)	1 (100)	0 (0.0)	1 (100)
**ES*β*L**	**6 (15.0)**	**6 (100)**	**6 (100)**	**6 (100)**	**6 (100)**	**3 (50.0)**	**6 (100)**	**6 (100)**	**4 (66.7)**	**5 (83.3)**	**5 (83.3)**	**4 (66.7)**	**5 (83.3)**	**5 (83.3)**	**3 (50.0)**	**4 (66.7)**	**5 (83.3)**	**5 (83.3)**	**4 (66.7)**	**5 (83.3)**	**3 (50.0)**	**2 (33.3)**	**3 (50.0)**	**6 (100)**
*E. cloacae*	1 (16.7)	1 (100)	1 (100)	1 (100)	1 (100)	1 (100)	1 (100)	1 (100)	1 (100)	1 (100)	1 (100)	1 (100)	1 (100)	1 (100)	1 (100)	1 (100)	1 (100)	1 (100)	1 (100)	1 (100)	1 (100)	1 (100)	0 (0.0)	1 (100)
*P. mirabilis*	3 (50.0)	3 (100)	3 (100)	3 (100)	3 (100)	1 (33.3)	3 (100)	3 (100)	2 (66.7)	2 (66.7)	2 (66.7)	1 (33.3)	2 (66.7)	2 (66.7)	1 (33.3)	1 (33.3)	2 (66.7)	2 (66.7)	2 (66.7)	2 (66.7)	1 (33.3)	0 (0.0)	2 (33.3)	3 (100)
*S. marcescens*	1 (16.7)	1 (100)	1 (100)	1 (100)	1 (100)	1 (100)	1 (100)	1 (100)	1 (100)	1 (100)	1 (100)	1 (100)	1 (100)	1 (100)	1 (100)	1 (100)	1 (100)	1 (100)	1 (100)	0 (0.0)	1 (100)	1 (100)	1 (100)	1 (100)
*Y. intermedia*	1 (16.7)	1 (100)	1 (100.0)	1 (100)	1 (100)	0 (0.0)	1 (100)	1 (100)	0 (0.0)	1 (100)	1 (100)	1 (100)	1 (100)	1 (100)	0 (0.0)	1 (100)	1 (100)	1 (100)	0 (0.0)	0 (0.0)	0 (0.0)	0 (0.0)	0 (0.0)	1 (100)
**Carbapenemases**	**4 (10.0)**	**4 (100)**	**4 (100)**	**4 (100)**	**4 (100)**	**2 (50.0)**	**2 (50.0)**	**2 (50.0)**	**2 (50.0)**	**2 (50.0)**	**2 (50.0)**	**4 (100)**	**2 (50.0)**	**2 (50.0)**	**2 (50.0)**	**2 (50.0)**	**3 (75.0)**	**3 (75.0)**	**2 (50.0)**	**3 (75.0)**	**3 (75.0)**	**1 (25.0)**	**0 (0.0)**	**4 (100)**
*E. sakazakii*	1 (25.0)	1 (100)	1 (100)	1 (100)	1 (100)	1 (100)	1 (100)	1 (100)	1 (100)	1 (100)	1 (100)	1 (100)	1 (100)	1 (100)	1 (100)	1 (100)	1 (100)	1 (100)	1 (100)	1 (100)	1 (100)	1 (100)	0 (0.0)^a^	1 (100)
*E. coli*	1 (25.0)	1 (100)	1 (100)	1 (100)	1 (100)	0 (0.0)	0 (0.0)	0 (0.0)	0 (0.0)	0 (0.0)	0 (0.0)	1 (100)	0 (0.0)	0 (0.0)	0 (0.0)	0 (0.0)	1 (100)	1 (100)	0 (0.0)	0 (0.0)	1 (100)	0 (0.0)^a^	0 (0.0)^a^	1 (100)
*K. pneumoniae*	1 (25.0)	1 (100)	1 (100)	1 (100)	1 (100)	0 (0.0)	0 (0.0)	1 (100)	0 (0.0)	0 (0.0)	0 (0.0)	1 (100)	0 (0.0)	0 (0.0)	0 (0.0)	0 (0.0)	0 (0.0)	0 (0.0)	0 (0.0)	1 (100)	1 (100)	0 (0.0)	0 (0.0)	1 (100)
*S. odorifera 1*	1 (25.0)	1 (100)	1 (100)	1 (100)	1 (100)	1 (100)	1 (100)	1 (100)	1 (100)	1 (100)	1 (100)	1 (100)	1 (100)	1 (100)	1 (100)	1 (100)	1 (100)	1 (100)	1 (100)	1 (100)	0 (0.0)	0 (0.0)	0 (0.0)^a^	1 (100)
***AmpC-*ES*β*L**	**23 (57.5)**	**23 (100)**	**23 (100)**	**23 (100)**	**23 (100)**	**19 (82.6)**	**23 (100)**	**23 (100)**	**23 (100)**	**23 (100)**	**23 (100)**	**20 (86.9)**	**19 (82.6)**	**17 (73.9)**	**13 (56.5)**	**11 (47.8)**	**18 (78.3)**	**18 (78.3)**	**20 (91.3)**	**11 (47.8)**	**1 2 (56.5)**	**16 (69.6)**	**20 (86.9)**	**13 (56.5)**
*C. koseri/farmeri*	1 (4.3)	1 (100)	1 (100)	1 (100)	1 (100)	1 (100)	1 (100)	1 (100)	1 (100)	1 (100)	1 (100)	1 (100)	1 (100)	1 (100)	1 (100)	1 (100)	1 (100)	1 (100)	1 (100)	1 (100)	0 (0.0)	1 (100)	1 (100)	1 (100)
*E. cloacae*	4 (17.4)	4 (100)	4 (100)	4 (100)	4 (100)	3 (75.0))	4 (100)	4 (100)	4 (100)	4 (100)	4 (100)	4 (100)	3 (75.0)	2 (50.0)	2 (50.0)	2 (50.0)	3 (75.0)	3 (75.0)	3 (75.0)	4 (10.0)	4 (100)	3 (75.0)	3 (75.0)	2 (50.0)
*E. sakazakii*	1 (4.3)	1 (100)	1 (100)	1 (100)	1 (100)	1 (100)	1 (100)	1 (100)	1 (100)	1 (100)	1 (100)	1 (100)	1 (100)	1 (100)	1 (100)	0 (0.0)	0 (0.0)	0 (0.0)	1 (100)	1 (100)	1 (100)	1 (100)	1 (100)	1 (100)
*E. coli*	3 (13.0)	3 (100)	3 (100)	3 (100)	3 (100)	1 (33.3)	3 (100)	3 (100)	3 (100)	3 (100)	2 (66.7)	2 (66.7)	2 (66.7)	3 (100)	2 (66.7)	3 (100)	3 (100)	3 (100))	3 (100)	1 (33.3)	2 (66.7)	1 (33.3)	1 (33.3)	1 (33.3)
*K. oxytoca*	1 (4.3)	1 (100)	1 (100)	1 (100)	1 (100)	0 (0.0)	1 (100)	1 (100)	1 (100)	1 (100)	1 (100)	1 (100)	1 (100)	1 (100)	1 (100)	0 (0.0)	1 (100)	1 (100)	1 (100)	0 (0.0)	0 (0.0)	0 (0.0)	1 (100)	0 (0.0)
*K. pneumoniae*	3 (13.0)	3 (100)	3 (100)	3 (100)	3 (100)	3 (100)	3 (100)	3 (100)	3 (100)	3 (100)	3 (100)	1 (33.3)	3 (100)	1 (33.3)	0 (0.0)	2 (66.7)	3 (100)	3 (100)	3 (100)	0 (0.0)	2 (66.7)	1 (33.3)	3 (66.7)	3 (66.7)
*Kluyvera* spp	5 (21.7)	5 (100)	5 (100)	5 (100)	5 (100)	5 (100)	5 (100)	5 (100)	5 (100)	5 (100)	5 (100)	5 (100)	4 (80.0)	4 (80.0)	4 (80.0)	2 (40.0)	3 (60.0)	3 (60.0)	4 (80.0)	1 (20.0)	1 (20.0)	4 (80.0)	5 (100)	2 (40.0)
*R. ornithinolytica*	1 (4.3)	1 (100)	1 (100)	1 (100)	1 (100)	1 (100)	1 (100)	1 (100)	1 (100)	1 (100)	1 (100)	1 (100)	1 (100)	1 (100)	1 (100)	0 (0.0)	1 (100)	1 (100)	1 (100)	1 (100)	0 (0.0)	1 (100)	1 (100)	0 (0.0)
*S. fonticola*	2 (8.7)	2 (100)	2 (100)	2 (100)	2 (100)	2 (100)	2 (100)	2 (100)	2 (100)	2 (100)	2 (100)	2 (100)	1 (50.0)	1 (50.0)	0 (0.0)	1 (50.0)	1 (50.0)	1 (50.0)	1 (50.0)	2 (100)	2 (100)	2 (100)	2 (100)	1 (50.0)
*S. odorifera 1*	2 (8.7)	2 (10.0)	2 (100)	2 (100)	2 (100)	2 (100)	2 (100)	2 (100)	2 (100)	2 (100)	2 (100)	2 (100)	2 (100)	2 (100)	1 (50.0)	0 (0.0)	2 (100)	2 (100)	2 (100)	0 (0.0)	0 (0.0)	2 (100)	2 (100)	2 (100)
**Total**	**40 (100)**	**40 (100)**	**40 (100)**	**40 (100)**	**40 (100)**	**28 (70.0)**	**38 (95.0)**	**38 (95.0)**	**36 (90.0)**	**37 (92.5)**	**37 (92.5)**	**33 (82.5)**	**32 (80.0)**	**27 (67.5)**	**21 (52.5)**	**20 (50.0)**	**30 (75.0)**	**30 (75.0)**	**32 (85.0)**	**30 (75.0)**	**23 (57.5)**	**23 (57.5)**	**26 (65.0)**	**25 (62.5)**

^a^strains presenting an intermediate phenotype (I) to the antibiotic.

Abbreviations: AKN, amikacin; AMC, amoxicillin + clavulanate; AMP, ampicillin; *C*. *koseri/farmeri*, *Citrobacter koseri/farmeri*; CAZ, ceftazidim; CFM, cefixim; CFT, cefolatine; CIP, ciprofloxacin; CS, colistin; CXT, cefoxitin; *E*. *Cloacae*, *Enterobacter cloacae*; *E*. *coli*, *Escherichia coli*; *E*. *gergoviae*, *Enterobacter gergoviae*; *E*. *sakazakii*, *Enterobacter sakasakii*; ERT, ertapenem; FEP, cefepime; FOS, fosfomycin; FUR, nitrofurantoin; GEN, gentamicin; IMI, imipenem; *K*. *oxytoca*, *Klebsiella oxytoca*; *K*. *pneumoniae*, *Klebsiella pneumoniae*; LVX, levofloxacin; OFL, ofloxacin; *P. mirabilis*, *Proteus mirabilis*; PIC, piperacillin; *R*. *ornithinolytica*, *Raoultella ornithinolytica*; *S*. *fonticola*, *Serratia fonticola*; *S*. *marcescens*, *Serratia marcescens*; *S*. *odorifera 1*, *Serratia odorifera 1*; TIC, ticarcillin; TOB, tobramycin; TSU, co‐trimoxazole; TZP, piperacillin + tazobactam; *Y. intermedia, Yersinia intermedia.*

Among the 40 strains of BS*β*L‐En screened for the susceptibility tests, resistance rates of 81.8*%* ± 0.16*%* and 61.7*%* ± 0.04*%* were obtained for the usual antibiotics and those of last resort. The different resistance profiles of the BS*β*L‐En are shown in Table 5.

Analysis of the results presented in Table 5 showed that all the strains were resistant to AMP, TIC and PIC with resistance rates of 100.0%. These rates varied in the presence of *β*‐lactamase inhibitors (AMC and TZP). The overall resistance rate was 70.0% for TZP and 100.0% for the AMC. High resistance rates to cephalosporins were also observed, including cephalotin (95.0%), cefixime (95.0%), cefoxitin (90.0%), cefotaxime (92.5%), ceftazidime (92.5%), and FEP (82.5%). Similarly, high resistance rates were recorded for aminoglycosides, with 80.0%, 67.5%, and 52.5% for gentamicin, tobramycin, and amikacin, respectively. Comparable resistance patterns were observed for other antibiotic classes, including cotrimoxazole (85.0%), ofloxacin (75.0%), ciprofloxacin (75.0%), fosfomycin (75.0%), nitrofurantoin (57.5%), and levofloxacin (50.0%). Finally, resistance to last‐resort antibiotics was also notable, with rates of 57.5% for imipenem, 62.5% for colistin, and 65.0% for ertapenem.

#### 3.1.4. Molecular Typing of BS*β*L‐En Strains and Characterization of Resistance Genes

Of the 40 presumptive BS*β*L‐En strains, 35 (87.5%) were positive for the *bla* genes screened. The various Enterobacterales producing these genes are shown in Table 6.

**Table 6:** Distribution of BS*β*L types, carbapenem and colistin resistance genes, as well as associations between ES*β*L/resistance genes according to bacterial strains.

Strains		BS*β*L								Molecular prevalence of resistance		
	Number of isolates	CTX‐M	TEM	IMP	TEM/CTX‐M	IMP/CTX‐M	IMP/TEM	CARBA/CTX‐M	CARBA/TEM/CTX‐M	IMP	CARBA	Mcr1/2
	*n*/*N*	*n*/*N*	*n* ^′^/*N*	*n*/*N*	*n*/*N*	*n*/*N*	*n*/*N*	*n*/*N*	*n*/*N*	*n*/*N*	*n*/*N*	*n*/*N*
*Citrobacter koseri/farmeri*	0/1 (0, 0)	—	—		—	—	—	—	—	—	—	—
*Enterobacter cloacae*	8/8 (100.0)	5/8 (62.5)	—		—	1/8 (12.5)	1/8 (12.5)	1/8 (12.5)	—	2/8 (25.0)	1/8 (12.5)	—
*Enterobacter gergoviae*	1/1 (100.0)	—	—		—	—	—	—	1/1 (100.0)	—	1/1 (100)	—
*Enterobacter sakasakii*	1/2 (50.0)	1/1 (50.0)	—		—	—	—	—	—	—	—	—
*Escherichia coli*	6/6 (100.0)	—	3/6 (50.0)	1/6 (16.7)		1/6 (33.33)	—		1/6 (16.66)	2/5 (40.0)	1/5 (20.0)	—
*Klebsiella oxytoca*	1/1 (100.0)	—	—		—	1/1 (100.0)	—	—	—	1/1 (100.0)	—	—
*Klebsiella pneumoniae*	4/4 (100.0)	2/4 (50.0)	—		2/4 (50.0)	—	—	—	—	—	—	—
*Kluyvera* spp	5/5 (100.0)	2/5 (40.0)	—		1/5 (20.0)	1/5 (20.0)	—		1/5 (20.0)	1/5 (20.0)	1/5 (20.0)	—
*Proteus mirabilis*	1/5 (25.0)	—	—		—	—	—	1/5 (20.0)	—	—	1/4 (25.0)	—
*Raoultella ornithinolytica*	1/1 (100.0)	—	—		—	—	—	1/1 (100.0)	—	—	1/1 (100.0)	—
*Serratia fonticola*	2/2 (100.0)	1/2 (50.0)	—		—	1/2 (50.0)	—	—	—	1/2 (50.0)	—	—
*Serratia marcescens*	1/1 (100.0)	—	—			—	—	—	1/1 (100.0)	—	1/1 (100.0)	—
*Serratia odorifera 1*	3/3 (100.0)	2/3 (66.7)	—		—	—	—	1/3 (33.33)	—	—	1/3 (33.33)	—
*Yersinia intermedia*	1/1 (100.0)	1/1 (100.0)	—		—	—	—	—	—	—	—	—
**Total**	**35/40 (87.5)**	**14/35 (40.0)**	**3/35 (8.6)**	**1/35 (2.9)**	**3/35 (8.6)**	**5/35 (14.3)**	**1/35 (2.9)**	**4/35 (11.4)**	**4/35 (11.4)**	**7/40(17.5)**	**8/40(20.0)**	**0/40 (0.0)**

The analysis of the results in Table 6 shows that the *bla*_CTX-M_ genes were detected in 31 strains (88.6%), of which 14 (40.0%) harbored *bla*_CTX-M_ alone, whereas the other 3 (8.6%) harbored *bla*_CTX-M_ in combination with *bla*_TEM_. However, 3 strains (8.6%) were positive for the *bla*_TEM_ gene (Table 6). Concerning the diversity of carbapenememases, the *bla*_CARBA_ and *bla*_IMP_ genes were detected in 15 (42.935%) strains. Only one strain (2.9%) harbored *bla*_IMP_ alone, whereas the other 5 (14.3%) harbored *bla*_IMP_ in association with *bla*_CTX-M_ and 2 others the *bla*_IMP-TEM_ genes. The *bla*_CARBA_ gene was only detected in association with *bla*_CTX-M_ (11.4%) alone or with *bla*_TEM_ (11.4%).

Furthermore, of the 40 strains that were molecularly screened, 15 (37.5%) possessed carbapenem resistance genes and none harbor colistin resistance genes.

#### 3.1.5. Correlation Between BS*β*L Typing and Phenotypic Resistance Profiles

Overall, kappa statistics indicated a lack of substantial agreement between the presence of BSBL‐producing Enterobacterales and antimicrobial susceptibility profiles. However, statistically significant associations were observed, particularly within the AmpC–ESBL co‐producing group. For this profile, agreement with imipenem was classified as moderate according to the Landis and Koch interpretation scale, with a kappa coefficient of 0.375 (95% CI: 0.12–0.63). In contrast, agreement with cefoxitin was considered fair, with a kappa value of 0.261 (95% CI: 0.01–0.51). Significant associations were also observed between this group and TZP as well as amikacin, suggesting specific phenotypic correlations despite overall heterogeneity in resistance patterns.

## 4. Discussion

The aim of this study was to describe the epidemiology of uropathogenic strains of BS*β*L‐En isolated in Libreville, Gabon, and to determine their resistance profiles to both first‐line and last‐resort antibiotics.

In the present study, UTIs diagnosed at the HIAOBO in Libreville between October and December 2019 were more frequent in women (70.4%). These results are similar to those reported in other studies [25–27]. Various factors predispose women to this type of infection, essentially their anatomical predisposition [25–27]. Indeed, the proximity between the genital and anal orifices in women facilitates the proliferation of germs toward the urethra which is also shorter [28].

This study included different age groups and it appears that most cases of UTIs were observed in patients aged 21 to 40 years (48.2%). These results are in agreement with those obtained from previous studies in Africa [25, 29]. They could be explained by the fact that subjects aged 20 to 40 have higher sexual activity [30]. The increased number of sexual partners and their capacity to check in hospital structures independently would explain their high number [28, 31, 32]. However, these results contrast with those reported in a study by Kot et al., [27] which showed that the rate of asymptomatic and symptomatic UTI was higher in patients in the age group of 60 years and older.

Partly the patients in this study came from a community setting (17.6%). This turnout can be explained by the fact that the hospital is the first place indicated for health care and for future patients seeking treatment or wishing to know their health status [31]. In addition, the cytobacteriological examination of urine (CBEU) is one of the most prescribed community examinations [33].

The overall prevalence of UTI in this study (18.31%) was comparable to those in Nepal [34], Libya [35], and Ethiopia [36], with 15.2%; 20.0% and 21.1%, respectively, but lower than those reported in Argentina, Saudi Arabia, and other regions of Gabon [14, 15, 37, 38]. The contrast observed between these results, in particular the low incidence in this study, may probably be explained by the epidemiological variability of UTIs, the differences in age, gender, the number of patients examined, the period of the study and the types of media chosen for the isolation the strains [31, 35]. In addition, the choice of the type of pathogens sought during this work, namely ES*β*L, compared to the study of Yala et al., [21] which only targeted. Enterobacterales responsible for UTIs in the same health care structure would also explain the low recorded prevalence [16].

Gram‐negative bacilli were the predominant uropathogens (81.5%), with Enterobacterales being the most frequently isolated group, consistent with previous literature [34, 39]. In addition, these Enterobacterales present resistance profile toward broad‐spectrum cephalosporins that seems to be at the origin of several types of infections [40, 41]. This resistance trait is mainly due to the production of *β*‐lactamases in particular, ES*β*Ls and AmpCs or even carbapenemase, which reduce therapeutic options [40, 41]. This study showed that 40/54 (74.1%) of the strains of Enterobacterales isolated from community UTIs are producers of *β*‐lactamases of the ES*β*L, AmpC and carbapenemase types. *E*. *cloacae* (14.8%), *E. coli* (11.1%), *Kluyvera* spp (9.3%) and *K. pneumoniae* (7.4%) species were the most common ones. These same uropathogens are also isolated in other studies although with a predominance of *E. coli* and *K. pneumoniae* [35, 42, 43]. These results also show that the prevalence of strains producing BS*β*L vary not only according to geographical areas, but also according to the types and species of uropathogens sought [44].

The present study recorded a phenotypic isolation rate of 72.5% of ES*β*L‐En. This frequency is similar to that obtained in the studies of Ugwu et al. [42] (96.0%) in Nigeria, Yala et al. [21] (61%) in Gabon, and much lower than that recorded in Gabon by Dikoumba et al. [45] (11.8%), Gebremichael et al. [46] (44.0%) in Ethiopia, and Kot et al. [27] (32.7%) in Poland. The *bla*_CTX-M_ gene is the most commonly found gene in 70.59% of cases. The high prevalence of CTX‐M ES*β*L corroborates the current epidemiological trend reported by numerous studies [21, 43, 45]. Indeed, CTX‐M enzymes currently overlap with other ES*β*Ls and have spread in the community environment, mainly due to their presence in highly mobilizable genetic areas of plasmids and transposons [47]. This expansion would probably be related to a widespread prescription of 3rd generation cephalosporins (cefotaxime and ceftriaxone) in treatments including UTIs [48, 49]. The data recorded largely corroborate the expansion of ES*β*L within strains of Enterobacterales in our country and in particular within uropathogens complicating the management of patients.

The high detection of BS*β*L would clearly be correlated with the co‐expression of ES*β*L‐AmpC enzymes of which 23 strains (57.5%) were produced in this study. In addition, 17.0% of the strains were also producing AmpC‐type *β*‐lactamases alone. The results obtained are comparable to those recorded by Perera et al. [50], whose prevalence was 19.0%. The co‐expression of ES*β*L and/or AmpC *β*‐lactamases is now identified as a major cause of resistance in species of the Enterobacterales family and seems to be related to the prescription of antibiotic therapy during the treatment of this infection [44, 51]. Furthermore, ES*β*L/AmpC genes are mostly located on mobile genetic elements such as plasmids, some of which are regarded as epidemic which would undoubtedly justify their presence [52].

Phenotypic carbapenemase production was observed in 10.0% of isolates, a rate comparable to studies from Egypt and Sri Lanka [50, 53], but higher than previous reports from Gabon (1.64%) [54]. Molecular analysis identified *bla_IMP_* and *bla*_CARBA_ genes although regional studies from Africa and Asia more commonly report *bla*_NDM_ and *bla*_OXA-48_ variants [53, 55]. Furthermore, comparison of our findings with those of Dikoumba et al. [54], whose isolates were also collected in Gabon, suggests geographic heterogeneity in carbapenemase distribution within Gabon in the various towns of Franceville vs. Libreville, Mouila, and Tchibanga.

The different BS*β*L isolates obtained showed resistance rates of 81.8% and 61.7%, respectively, for common antibiotics and those of last resort. The high rate of resistance to common antibiotics supports the current pattern of ES*β*L/AmpC‐En resistance to other antibiotic families [47, 51]. Although carbapenems remain the molecules of choice against these ES*β*L and AmpC producing strains, the nonprescription of these molecules would clearly complicate the treatment of UTIs due to the prevalence of BS*β*L‐En in our countries. In addition, therapeutic alternatives that include conventional combinations such as *β*‐lactam + *β*‐lactamase inhibitors, cephamycins, temocillin, aminoglycosides, tigecycline, and fosfomycin [51] do not seem to be judicious treatments in Gabon considering the high rates of resistance to fosfomycin (75.0%), and aminoglysosides, respectively, to gentamicin (80.0%), tobramycin (67.5%), and amikacin (52.5%) recorded in this study.

Moreover, carbapenems and colistin are considered last‐resort antibiotics for the treatment of BS*β*L‐En and knowledge of their susceptibility profiles is essential for optimal therapeutic management [56, 57]. Overall, these isolates exhibited a high resistance rate (61.7%) to last‐resort antibiotics. In this study, high levels of phenotypic resistance to ertapenem (65%) and imipenem (57.5%) was observed.

These findings are consistent with those reported in other studies, although the specific antibiotics tested differed within the same class. Indeed, Pandit et al., [34] reported resistance rates of 90.3% and 83.8% to imipenem and meropenem, respectively, likewise Krir et al., found 63.2% of ES*β*L producing isolate were resistant to imipenem [58]. These profiles would probably be justified by the association of a decrease in their membrane permeability and the production of ES*β*L and/or AmpCs as demonstrated in the literature [59]. In addition, the presence of carbapenemases is also correlated with this decrease or even loss of susceptibility to carbapenems [60, 61] of which 44.1% of ES*β*L are carriers, thus justifying these phenotypes obtained. Sporadic cases of colistin‐resistant isolates in both hospital and community settings [62] would warrant evaluation of the susceptibility of ES*β*L in this study to this antibiotic.

Sporadic cases of colistin‐resistant isolates in both hospital and community settings [62] justify the evaluation of the susceptibility of ES*β*L in this study to this antibiotic. The absence of *mcr*‐1 and *mcr*‐2 genes despite phenotypic colistin resistance (62.5%), suggests alternative mechanisms, including chromosomal mutations affecting regulatory systems such as pmrA/pmrB and phoP/phoQ, as well as lipid A modifications [59, 63]. Plasmid‐mediated resistance mechanisms surch as IncHI2 plasmid co‐transmissible and carriers of the *mcr-1* gene were not investigated in this study but could also contribute to resistance not only to colistin, cephalosporins and carbapenems [64]. Although the *mcr-1* and *mcr-2* genes were not detected in this study, this high phenotypic resistance to colistin could be attributed to production of ES*β*Ls and AmpC. Indeed, Gram‐negative bacteria producing ES*β*L, MBL and AmpC hosting the *mcr* genes on the same plasmid have been reported [65].

Furthermore, the fact that CTX‐M genes, naturally chromosomally mediated in Enterobacterales species, are easily transmitted by conjugation in vivo via a plasmid between them, would not only explain the high expression but also the emergence of this type of ES*β*L [66]. This would explain why it is the most frequently associated in this study (66.7%; *n* = 10/15) compared to TEM‐type ES*β*L (6.7%; *n* = 1/15). At the same time, the high levels would be explained by the association or cohabitation of *bla* genes within strains that would promote ES*β*L resistance to last‐line antibiotics [67].

Finally, limitations of this study include the restricted molecular characterization of ES*β*L genes, the absence of AmpC, carbapenemase, and *mcr* genes panels beyond selected targets. Furthermore, the use of antibiotic‐supplemented culture media at concentrations above standard recommendations may have influenced bacterial recovery rates.

## 5. Conclusion

In conclusion, this study shows that BS*β*L‐En are involved in UTIs in Libreville, Gabon with the two prominent species being *E. cloacae* and *E. coli*. These strains have high prevalence rates of production of BS*β*L of which the most predominant are *β*‐lactamases of the AmpC and ES*β*L types. The most common genetic support for BS*β*L is CTX‐M type. In addition, these BS*β*L‐En show high rates of resistance to both first‐ and last‐line therapeutic antibiotics. The presence of *bla*_IMP_ and *bla*_CARBA_ genes confirms the genetic support of carbapenem resistance and circulating carbapenemases.

Given these concerning findings, strengthened surveillance of uropathogens through systematic screening of clinical isolates and continuous monitoring of antimicrobial resistance trends is essential at both local and national levels.

**Nomenclature**
AKN: amikacin
AMC: amoxicillin + clavulanate
AMP: ampicillin
API: Analysis of Identification Profiles
AZT: aztreonam
BCC: heart–brain agar
BS*β*L‐En: broad‐spectrum beta‐lactamase‐producing Enterobacterales
CA‐SFM: Antibiogram Community of the French Microbiology Society
CAZ: ceftazidime
CFM: cefixime
CFT: cefolatine
CIP: ciprofloxacin
CS: colistin
CTX: cefotaxime
CXT: cefoxitin
DNA: deoxyribonucleic acid
EAS: emergency ambulatory service
ERT: ertapenem
ES*β*L: extended‐Spectrum *β*‐lactamase
ES*β*L‐En: extended‐spectrum *β*‐lactamase‐producing Enterobacterales
FEP: cefepime
FOS: fosfomycin
FOX: cefoxitin
FUR: nitrofurantoin.
GEN: gentamicin
HIAOBO: Hôpital D′Instruction des Armées Omar Bongo Ondimba
IMI: Imipenem
LABMC: Laboratory of Molecular and Cellular Biology
LVX: levofloxacin
McC‐CTX: MacConkey agar supplemented with cefotaxime
MIC: minimal inhibitory concentration
OFL: ofloxacin
PCR: polymerase chain reaction
PIC: piperacillin
TBE: Tris‐borate‐EDTA
TIC: ticarcillin
TOB: tobramycin
TSU: co‐trimoxazole
TZP: piperacillin + tazobactam
USTM: Masuku University of Sciences and Technology
UTIs: urogenital tract infections

## Author Contributions

All authors contributed to the study conception and design. Material preparation, data collection, and analysis were performed by Ornella Zong Minko, Jean Fabrice Yala, Rolande Mabika Mabika, and Franck Mounioko. The technical platform and sample collection site were provided by Anicet C. Dikoumba and Elvire Anita Mbongo‐Kama. The first draft of the manuscript was written by Ornella Zong Minko, Rolande Mabika, Jean Fabrice Yala, and Alain SOUZA. And all authors commented on previous versions of the manuscript.

## Funding

No funding was received for this manuscript.

## Disclosure

All authors read and approved the final manuscript.

## Ethics Statement

The study was approved by the Gabonese National Ethics Committee for Research and the Ministry of Health (PROT N° 0020/2015/SG/CNE) and was conducted in accordance with the ethical standards of the 1964 Declaration of Helsinki.

## Conflicts of Interest

The authors declare no conflicts of interest.

## Data Availability

The data that support the findings of this study are openly available in BioStudies at https://www.ebi.ac.uk/biostudies/studies/S-BSST1054, reference number:S‐BSST1054.
